# Common Mental Disorders Among Informal Primary Caregivers of Adults With Mental Illness During the Coronavirus Disease 2019 Epidemic in Eastern Ethiopia: A Cross-Sectional Study

**DOI:** 10.3389/fpsyt.2021.676379

**Published:** 2021-08-23

**Authors:** Tilahun Abdeta, Defaru Desalegn

**Affiliations:** ^1^Department of Psychiatry, School of Nursing and Midwifery, College of Health and Medical Sciences, Haramaya University, Harar, Ethiopia; ^2^Department of Psychiatry, Faculty of Public and Medical Science, Mettu University, Mettu, Ethiopia

**Keywords:** Ethiopia, prevalence, associated factors, caregivers, common mental disorders

## Abstract

**Background:** Coronaviruses (CoVs) are emerging respiratory viruses and cause illnesses ranging from the common cold to severe acute respiratory syndrome (SARS). Informal primary caregivers of individuals with mental illness were special populations suffering from both the burden of caring for mentally ill individuals and the danger of the Coronavirus disease 2019 (Covid-19) epidemic.

**Objective:** The objective of the study was to assess the prevalence and associated factors of common mental disorders (CMDs) among the informal primary caregivers of adults with mental illness during the Covid-19 epidemic.

**Methods:** A hospital-based cross-sectional study design was conducted from July 1 to 30, 2020. A systematic random sampling technique was used to get samples of informal primary caregivers. Data were analyzed by bivariable and multivariable logistic regression analysis. In the final model, variables having a *p*-value < 0.05 were declared as associated with CMDs.

**Result:** Out of a total of 218 informal primary caregivers, 215 responded to this study. The prevalence of CMDs was 40.5% [95% confidence interval (CI) = 36.66, 44.3%] among informal primary caregivers. Being female informal primary caregivers [adjusted odds ratios (AOR) 1.98, 95% CI = 1.05, 3.76], being student informal primary caregivers (AOR 5.8, 95% CI: 1.2, 28.4), caring patients with psychosis (AOR 3.33, 95% CI: 1.12, 9.92) and caring patients with bipolar disorder (AOR 3.12, 95% CI: 1.35, 7.23) were significantly associated with CMDs.

**Conclusion:** Our study cannot show the causal relationship due to its cross-sectional study design nature. However, this study showed relatively higher prevalence of CMDs among informal primary caregivers of adults with any mental illness during the Covid-19 epidemics relative to pre-Covid-19 times. Being a female caregiver, being a student caregiver, and caring for adults with psychosis and bipolar disorder were factors associated with CMDs. Attention should be given, and essential psychosocial care should be provided, to maintain the mental health of informal primary caregivers of individuals with mental illness especially during the Covid-19 pandemic.

## Introduction

Since December 2019, the world is fighting an outbreak of a novel infectious disease known as Coronaviruses disease 2019 (Covid-19) (1). Coronaviruses (CoVs) are emerging respiratory viruses and cause illnesses ranging from the common cold to severe acute respiratory syndrome (SARS) (2). Covid-19 was detected for the first time in December 2019, like a viral outbreak in Wuhan City of Central Hubei Province of China, and on December 31, 2019, it was announced to the World Health Organization (WHO) (3). In Ethiopia, on March 13, 2020, the Federal Ministry of Health has confirmed and announced the first Covid-19 case in Addis Ababa, and on April 5, 2020, the first death of a Covid-19 patient, a 60-year old woman was reported in Addis Ababa (4).

The Covid-19 pandemic has an impact on physical health, psychological and social aspects, and could affect the mental health status of the population (5). As Covid-19 is an emerging disease and is having the most devastating effects globally, it can cause community mental health problems (6). An online study in Spain showed that the Covid-19 pandemic had added to caregiver stress and pain (7). The number of caregivers with mental distress during Covid-19 epidemics was more than double than reported before the Covid-19 epidemics (8). The pandemic could lead to a significant decline in family functioning triggered by the incredible pressure imposed by Covid-19 limits and precautions; in particular, the increased pressure on caregivers might result in an imbalance in the demand for resources and disagreements between family members (9). Having a mental illness during a global pandemic could have a huge effect on the entire family members including financially, socially, and interpersonally, especially when combined with the impact of larger-scale effects of the pandemic on socioeconomic circumstances (10).

Mental disorders accounted for about 12.3% of the global burden of disease in 2001 (11). Depression and anxiety disorders are known as “common mental disorders” (CMDs) (11, 12). In the United States National Comorbidity Survey, the prevalence of mood and anxiety disorders was 20.8 and 28.8%, respectively (13). Available community-based studies in Ethiopia conducted in Butajira and Kombolcha towns and Addis Ababa City revealed that the magnitude of CMDs ranges from 17 to 32.4% (14).

Informal primary caregivers are caregivers who deliver unpaid care to somebody with whom they have a personal connection (15). Many individuals caring for relatives with mental disorder feel stigmatized (16). Families provide not only personal care, including eating, bathing, and giving drugs, but also give emotive support to their relatives with mental health problems (17). Caregivers usually experience substantial physical and mental problems due to lack of financial, personal, emotional support, stigma, and ignorance of their health (18, 19). Individuals with a severe mental disorder such as severe depression are unable to fulfill daily roles in the society normally expected of individuals of their age and intellectual ability; thus, they are most likely getting family care (20). Additionally, they often engage in difficult behaviors, and caregiver relatives are responsible of managing these behaviors (21).

Many studies showed that informal caregivers of individuals with mental disorders suffer from mental distress and get inadequate help from mental health professionals. Even in developed countries, only 10% of informal caregivers gets psychological treatments (22). In Ethiopia, there is no treatment guideline/policy specifically established to provide prevention/treatment service for informal primary caregivers of persons with mental disorders unless they come to health institutions seeking help after being ill. Even if there is scarce research on the effect of Covid-19 on caregivers (5), there are many previous studies conducted before the Covid-19 pandemic on CMDs among the primary informal caregivers of adult individuals with mental illness. A British research reported that mental distress was relatively two times higher among caregivers of mentally ill individuals (36%) than in the general population (20.8%) (23, 24). A pre-Covid-19 study in Ethiopia showed that the overall magnitude of mental distress among informal primary caregivers of individuals with a severe mental disorder was 56.7% (25). Also, another pre-Covid-19 study in Ethiopia reported that the prevalence of depression among primary caregivers of patients with severe mental illness was 19% (26). Besides, the prevalence of mental distress was found to be 27.1% among caregivers of patients with epilepsy in Ethiopia during the pre-Covid-19 period (27).

Different pre-Covid-19 studies showed that factors including duration of illness, patients having more than one episode of suicidal attempts, patients having behavioral problems, duration and type of care given, joblessness, cognitive and functional disabilities of care recipient, perceived stigma, negative caregiving judgment, loneliness, irritation, sadness, guilty feeling, shamefulness in caring mentally ill persons, unsatisfactory social support, age of patient, poor coping, and secondary stressors, including family conflict and finances, were significantly associated with psychological distress among primary caregivers of individuals with mental illness (17, 28). Other studies conducted among the general population revealed that pre-existing mental health conditions, increased smoking and alcohol drinking over the last 4 weeks, high levels of fear, and being female were associated with higher levels of psychological distress during the Covid-19 pandemic (29–31). Generally, the burden of the caregivers affects the patients, caregivers themselves, other family members, and health care systems. The poor quality of life of caregivers could cause behaving violently to the patients and poor caring, which can result in the relapse of the patients (20).

Recently, only a few studies have focused on the psychological health of informal primary caregivers of persons with mental disorders during pre-Covid-19 times in Ethiopia (25–27). To our best knowledge, there is no study conducted on the psychological health of informal primary caregivers of persons with mental disorders in Ethiopia during the Covid-19 epidemic. Even the few available studies conducted pre-Covid-19 epidemic time in Ethiopia were focused on caregivers of individuals with severe mental disorders, and the independent variables they included were not comprehensive enough. For instance, in the current study, we have included many variables (age of caregivers, age of patients, average monthly income, duration of illness in a year, time spent with patient per day, family history of mental illness and suicide, and history of suicide attempt of patient), which were not included in previously available studies in Ethiopia (25–27). Therefore, our study pointed to fill the gap as we conducted on the prevalence and associated factors of CMDs among the informal primary caregivers of adults with mental illness during the Covid-19 epidemic and who were attending mental health care service at Hiwot Fana Specialized University Hospital (HFSUH), Eastern Ethiopia. The result of this research will serve as base information to initiate giving necessary service for key informal caregivers of persons with mental illness including routine screening/assessment and give early intervention, especially during this Covid-19 pandemic period.

## Materials and Methods

### Study Setting

We conducted this study from July 1 to 30, 2020 among the informal primary caregivers of adults with mental disorders and attending mental health care service at Hiwot Fana specialized University Hospital (HFSUH), Harar town, Eastern Ethiopia. Harar is located 527 km away from Addis Ababa in the eastern direction. In Harar town, there are 1 University teaching hospital, 3 government hospitals, 2 private hospitals, 8 health centers, 19 health posts, and 1 FGAE (Family Guidance Association of Ethiopia) clinic. The HFSUH is one of the oldest governmental hospitals, established in 1941 E.C. (32) and provides different clinical services including the psychiatry unit. The psychiatric clinic has 10 beds for inpatients and four rooms for outpatient services. Annually, more than 5,000 children and adult patients get service at both the outpatient and inpatient departments of the psychiatry clinic.

### Study Design

The hospital-based quantitative cross-sectional study design was employed.

#### Source Population

All informal primary caregivers of adults with any mental illness and attending psychiatry clinic unit (outpatient and inpatient levels) at HFSUH were considered as a source of population.

#### Study Population

Sampled informal primary caregivers of adults with any mental illness and attending psychiatry clinic unit (outpatient and inpatient level) at HFSUH during the data collection period were considered as a study population. Informal primary caregivers of above 18 years were included, while those who have psychiatric disorder history before being a caregiver, professional caregivers, and who were missing patients for at least 1 month during the last 3 months were excluded.

#### Sample Size Determination

Single population proportion formula (33) was used to calculate the sample size by considering the assumptions: Z = the standard normal distribution (Z = 1.96), with confidence interval (CI) of 95%, *P* = the prevalence of CMDs among primary informal caregivers of adult persons with a mental disorder reported to be 56.7 % by a study conducted in Ethiopia, Addis Ababa (25), d = the margin of error = 5%.

Then using the above assumptions, the sample size was 377 subjects. However, because the total population in this study was <10,000, we used the finite population correction formula (33) to calculate the final sample size, and we get 198 subjects.

$$\text{nf}=\frac{ni}{\left(1+\left(\frac{ni-1}{N}\;\right)\right)}$$

where

nf = the final sample size
ni = the initial sample size calculated above (377)
N = the average total number of primary informal caregivers of adult individuals with mental disorders and attending mental health care service at HFSUH per month (416)

$$\text{nf=}\frac{\text{377}}{\left(\text{1+}\left(\frac{\text{377-1}}{\text{416}}\right)\right)}=\;198$$

Finally, by adding 10% (198 × 0.10 = 20) non-response rate, the final sample size was 198 + 20 = 218.

### Sampling Technique

The systematic random sampling technique (34) was used to select study participants from HFSUH inpatient and outpatient departments. The average number of primary informal caregivers of adult individuals with any mental disorders and attending mental health care service at HFSUH per month was reviewed, and about **416** caregivers (**122** for inpatients and **294** for outpatients) were eligible for this study.

The sampling interval for this study was calculated by the sampling interval calculation formula (34).

$$\begin{array}{l} \text{Sampling interval}=\frac{\text{total number of basic sampling units in the population }\left(\text{N}\right)}{\text{number of sampling units needed for the sample }\left(\text{n}\right)} \end{array}$$

Therefore, the sampling interval = 416/218 = 2.

This means that the first primary caregiver included in the study was selected by the lottery method. Then we continued to select one primary informal caregiver from every subsequent group of 2 until the required sample size was reached (n = 218).

The final sample size (n = 218) was allocated to each unit of a hospital, n1 = 64 primary informal caregivers from the inpatient unit and n2 = 154 primary informal caregivers from the outpatient department using the proportional allocation formula.

$$\text{Proportional allocation formula}:n_{\text{x}}=\frac{n.\;Nx}{NT}$$

where

**N** = the average total number of primary informal caregivers (416)
**N1** = the total number of primary informal caregivers of inpatients (122)
**N2** = the total number of primary informal caregivers of outpatients (294)
**n** = the final total sample size (218)
**n1** = the sample proportionally allocated to primary caregivers of inpatients
**n2** = the sample proportionally allocated to primary caregivers of outpatients

Therefore,

$$\begin{array}{lll} n_{1}=\frac{218.122}{416} & = & \;\text{64}\\ n_{2}=\frac{218.294}{416} & = & \;\text{154} \end{array}$$

### Data Collection

Data were collected through face-to-face interviewing and reviewing the medical records of the patients. The interview was conducted using the interviewer-administered standardized questionnaire adopted after extensive literature review (25, 26, 35). The questionnaire comprises sociodemographic variables, substance use-related questions, patient-related factors, SRQ-20 items to assess the level of CMDs of informal primary caregivers, Oslo Social Support Scale (OSSS-3) to assess the perceived social support of the caregivers, and family interview schedule (FIS) instrument to assess the perceived stigma of the caregivers. The questionnaire was first developed in English and then translated into the local Amharic and Afaan Oromo languages, and back translation into English was undertaken. However, some of the illness-related factors of the patients, including the total duration of illness in a year, number of admissions, and history of suicidal attempt of patients, were obtained from the medical records of the patients.

Data were collected by five BSc psychiatric nurses and supervised by three MSc psychiatry professional supervisors and principal investigators. All data collectors and supervisors proceeded with the data collection by keeping a 2- meter social distancing and wearing a protective face mask and a surgical glove. We assessed the CMDs of the primary caregiver using the Self-Reported Questionnaire 20 items (SRQ – 20), which is a 20 – item screening tool settled by the World Health Organization (WHO) to detect suspected CMDs cases (36). This tool has 20 items scored 0 or 1, and the questionnaire was also validated in Ethiopia, with sensitivity (85.7%) and specificity (75.6%) (37). A score of 0 shows that the symptom was absent throughout the last month, and a score of 1 shows the symptom was present. The SRQ – 20 has different cutting points in community based and institution based, and usually, a cut-point of ≥7 is used in institution based (37). Even if SRQ − 20 was designed as a self-administered scale, due to the low literacy rate in unindustrialized countries, this tool was suitable for an interviewer-administered questionnaire (38).

We used the Oslo 3 − item Social Support Scale (OSS − 3) to assess the social support of the caregivers. The OSS − 3 scores ranged from 3 to 14 with a score of 3 − 8 = poor support, 9 − 11 = intermediate support, and 12 − 14 = strong support. This tool was validated in African countries (39), and this tool was also used in previous Ethiopian studies (40).

Data about caregivers' perceived stigma were collected using the stigma part of a family interview schedule (FIS) instrument (with Cronbach's alpha = 0.85), which was prepared by the WHO on the outcome and course of schizophrenia and stigma (35), and this tool was used in a previous Ethiopian study. The stigma part has 14 items. Each stigma question was rated as a four-point scale from “not at all” rated 0, “sometimes” rated 1, “often” rated 2 to “a lot” rated 3. To assess the distribution of perceived stigma responses among groups, a stigma sum score was calculated by adding all positive responses (>0) for each of the 14 items, and we dichotomized to “yes/no” assuming caregivers who scored positive responses (>0) for at least one of the 14 stigma items as having perceived stigma “yes” and who scored 0 for all stigma items as not having perceived stigma “no”.

We obtained informed written consent from each study participant after telling them the objective of our study. We had informed the study participants as they have the right to ask for any unclear questions. Their data were kept confidential, and we did not document their names.

### Study Variables

#### Dependent Variable

Status of CMDs (yes/no).

#### Independent Variables

**Sociodemographic Variables** (age of caregiver and age of the patient, sex of caregivers, marital status, educational status, occupational status, and average monthly income), *clinical factors* (diagnosis of patients, duration of care given, time spent with the patient per day, number of admissions, duration of illness of patient, family history of mental illness of caregivers, history of suicide attempt of patient, and family history of suicide of caregivers), *substance-related variables* (current and lifetime substance use), *perceived stigma*, and *perceived social support*.

### Data Processing and Analyses

We first checked for collected data completeness and codes. We used EpiData (version 3.1) software to import data and SPSS (version 25) statistical software for statistical analyses. Descriptive analyses were used to describe the variables (frequencies, percentages, and tables). Bivariable and multivariable logistic regression analyses were performed to identify the factors associated with the CMDs. All variables with a *p*-value of < 0.25 in the bivariable analyses were entered into multivariable logistic regression to control potential confounders. We used a *p*-value < 0.25 in bivariable analyses to give a large chance to variables and not to miss important variables, which might have weak association in bivariable and could have strong association after adjusted with other variables. Then factors with a *p*-value of < 0.05 were finally considered to be significantly associated with CMDs, and the adjusted odds ratio (AOR) with 95% confidence interval (CI) was used.

### Data Quality Control

A pre-test was conducted on 11 primary informal caregivers (5% of the total sample size), and necessary amendments were made. We used 5% of the sample size because all of the study participants have similar language and cultural characteristics, and we assumed that 5% of the sample size can uncover common problems. During data collection process, the questionnaire was checked for its completeness on a daily basis by the principal investigators and supervisors. Prior to the data collection time, both data collectors and supervisors were given a 1-day training about the study objectives and data collection procedures.

### Ethical Considerations

We obtained ethical clearance from the Institutional Health Research Ethics Review Committee (IHRERC) of Haramaya University, College of Health and Medical Sciences. We had explained the study purpose to each participant before their participation. A formal permission letter was obtained from the administrative department of the hospital. Written and signed informed consent was obtained from the participants for their voluntary participation. Confidentiality was maintained by omitting personal identifications.

## Result

### Sociodemographic Characteristics of Participants and Patients

Out of 218 total primary caregivers, 215 respondents enrolled in this study, providing a response rate of 98.62%. The remaining three (3) individuals refused to participate in the study for unknown reasons. The mean age of respondents was 35 with standard deviation (SD) = 1.62 years old ranging from 18 to 70 years, and 55.8% (n = 120) were female, and 63.7% (n = 137) were married. The 23.3 % (n = 50) of the respondents learned up to secondary school, and 29.8% (n = 64) were farmers. Besides, 43.30% (n = 93) of the participants have an average monthly income of <1,000 Ethiopian birrs (ETB), and 47.40% (n = 102) have intermediate perceived social support (Table 1).

**Table 1:** Sociodemographic characteristics of the primary informal caregivers of adults with mental illness at Hiwot Fana Specialized University Hospital (HFSUH) (*n* = 215), Ethiopia, 2020.

**Sociodemographic variables**	**Frequency (*N*)**	**Percent (%)**
**Sex of the caregivers**		
Male	95	44.20
Female	120	55.80
**Age of the caregivers**		
18–25	51	23.70
26–33	62	28.80
34–41	44	20.50
42–49	32	14.90
50 and above	26	12.10
**Age of the patients**		
18–25	70	32.60
26–33	60	27.90
34–41	52	24.20
42–49	33	15.3
**Marital status**		
Married	137	63.70
Single	61	28.40
Divorced	8	3.70
Others^*^	9	4.20
**Educational status**		
Unable to read and write	46	21.40
Able to read and write	24	11.20
Primary school	49	22.80
Secondary school	50	23.30
College and above	46	21.40
**Occupational status**		
Government employer	32	14.90
Merchant	49	22.80
Farmer	64	29.80
Student	14	6.50
Other^**^	56	26.00
**Average monthly income in Ethiopian Birr (ETB)**		
<1,000	93	43.30
1,001–3,000	43	20.00
>3,000	79	36.70
**Perceived social support**		
Poor support	73	34.00
Intermediate support	102	47.40
Strong support	40	18.60

* *Separated, widowed*.

** *Daily laborer, private business, housewife*.

### Clinical and Substance Use-Related Characteristics of Participants and Patients

Out of 215 study participants, 59.10% (n = 127) and 54.90% (n = 118) had used at least one of khat, cigarette, or alcohol in their lifetime and during the last 3 months, respectively. About half of the caregivers (49.30%, *n* = 106) had cared for patients for 1-month to 1-year duration and 41.90% (n = 90) of the primary caregivers spent the whole day with the patient. Out of the total samples of primary caregivers, 31.20% (n = 67) of their patients were ill for 1 − 3 years, and 54.90% (n = 118) of the patients have no admission history. The 37.20% (n = 80) of the patients were diagnosed with psychosis, and 20.90% (n = 45) of them had a history of the suicide attempt. Also, 19.10% (n = 41) and 11.60% (n = 25) of the primary informal caregivers had a family history of mental illness and suicide, respectively. Again, 17.20% (n = 37) of the caregivers reported perceived stigma (Table 2).

**Table 2:** Clinical and substance use-related characteristics of primary informal caregivers of adults with mental illness at the HFSUH (*n* = 215), Ethiopia, 2020.

**Sociodemographic variables**	**Frequency (*N*)**	**Percent (%)**
**Perceived stigma**		
Yes	37	17.20
No	178	82.80
**Duration of illness of patient**		
<1 year	49	22.80
1–3 years	67	31.20
4–6 years	47	21.90
7 years and above	52	24.20
**Duration of care given**		
<1 month	18	8.40
1 month to 1 year	106	49.30
2–3 years	37	17.20
4 years and above	54	25.10
**Time spent with patient per day**		
1–6 h	56	26.00
7–12 h	69	32.10
Whole day	90	41.90
**Number of admissions of patient**		
1 time	49	22.80
2 times and above	48	22.30
No admission	118	54.90
**Family history of mental illness of caregivers**		
Yes	41	19.10
No	174	80.90
**Family history of suicide of caregivers**		
Yes	25	11.60
No	190	88.40
**History of suicide attempt of patient**		
Yes	45	20.90
No	170	79.10
**Diagnosis of patients**		
Psychosis	80	37.20
Bipolar disorder	60	27.90
Depression	50	23.30
Anxiety disorder	25	11.60
**Lifetime substance use of at least one of khat, cigarette, or alcohol**		
Yes	127	59.10
No	88	40.90
**Substance use during the last 3 months of at least one of khat, cigarette, or alcohol**		
Yes	118	54.90
No	97	45.10

### Prevalence of Common Mental Disorder Among Informal Primary Caregivers

The prevalence of CMDs among 215 informal primary caregivers of adults with mental illness during the Covid-19 epidemic was 40.50%; 95% CI = 36.66, 44.34 that met the cutoff point, which is ≥7 of the SRQ − 20 for a CMDs. The magnitude of CMDs among female informal primary caregivers was 58.30% (*n* = 70). Similarly, 59.40% (*n* = 19) of informal primary caregivers who were found between the age group of 42 and 49 years had CMDs. More than half percent, 56.70% (*n* = 34), of the informal primary caregivers who had been giving care for patients found between the age group of 26 and 33 were found having CMDs. The prevalence of CMDs among the farmer primary caregivers was 62.50% (*n* = 40). Besides, the prevalence of CMDs among informal primary caregivers who had poor social support was found highest compared with primary caregivers who had strong social support (46.60 vs. 40%). The prevalence of CMDs among the informal primary caregivers who had ever used at least one of khat, cigarette, or alcohol was 58.30% (*n* = 74), while the prevalence of CMDs among informal primary caregivers who had used at least one of khat, cigarette, or alcohol during the past 3 months was 59.30% (*n* = 70). The magnitude of CMDs among the informal primary caregivers who had a family history of mental illness was higher than those who had no family history of mental illness (68.30 vs. 47.10%). The prevalence of CMDs among the informal primary caregivers of patients who were diagnosed with psychosis, bipolar disorder, depression, and anxiety disorders was 65.00% (*n* = 52), 48.30% (*n* = 29), 40.00% (*n* = 20), and 36.00% (*n* = 9), respectively.

### Bivariable Logistic Regression Analyses of Factors Associated With Common Mental Disorders Among Informal Primary Caregivers of Adults With Any Mental Illness

In our study, bivariable logistic regression analyses revealed that factors including being female caregivers, being student caregivers, lifetime substance use at least one of khat, cigarette, or alcohol, and substance use in the past 3 months at of least one of khat, cigarette, or alcohol, time spent with the patient per day, number of admissions of the patient, family history of mental illness of the caregiver, family history of suicide of the care giver, and diagnosis of the patient were found having significant association with CMDs among informal primary caregivers of adults with mental illness at *p*-value ≤ 0.25 (Table 3).

**Table 3:** Binary logistic regression analysis of factors associated with common mental disorders (CMDs) among primary informal caregivers of adults with mental illness at the HFSUH, Ethiopia, 2020.

**Variables**	**Common mental disorder**		***p*-value**	**Crude odds ratio (COR) (95% CI)**
	**Yes**	**No**		
**Sex of the caregivers**				
Male	50	45	Reference	Reference
Female	70	50	0.01	2.10 (1.18, 3.55)
**Age of the caregivers**				
18–25	25	26	0.58	0.76 (0.29, 1.98)
26–33	33	29	0.35	0.64 (0.26, 1.62)
34–41	22	22	0.53	0.73 (0.28, 1.95)
42–49	19	13	0.19	0.50 (0.18, 1.43)
50 and above	11	15	Reference	Reference
**Age of patients**				
18–25	30	40	Reference	Reference
26–33	34	26	0.08	0.54 (0.27, 1.12)
34–41	28	24	0.23	0.64 (0.31, 1.32)
42–49	17	16	0.41	0.71 (0.31, 1.62)
**Marital status**				
Married	67	70	Reference	Reference
Single	31	30	0.80	0.93 (0.51, 1.69)
Divorced	4	4	0.95	0.96 (0.23, 3.98)
Others^*^	8	1	0.48	0.12 (0.15, 9.83)
**Educational status**				
Unable to read and write	24	22	0.53	1.30 (0.57, 2.97)
Able to read and write	16	8	0.52	0.71 (0.25, 1.99)
Primary school	22	27	0.18	1.74 (0.77, 3.93)
Secondary school	21	29	0.10	1.96 (0.87, 4.42)
College and above	27	19	Reference	Reference
**Occupational status**				
Government employer	19	13	Reference	Reference
Merchant	20	29	0.11	2.12 (0.86, 5.25)
Farmer	40	24	0.77	0.88 (0.37, 2.18)
Student	3	11	0.02	5.36 (12.5, 23.04)
Other^**^	28	28	0.39	1.46 (0.61, 3.52)
**Average monthly income**				
<1,000	47	46	0.99	1.04 (0.55, 1.83)
1,001–3,000	23	20	0.76	0.89 (0.42, 1.88)
>3,000	40	39	Reference	Reference
**Perceived social support**				
Poor support	34	39	0.17	1.72 (7.90, 37.6)
Intermediate support	40	62	0.33	1.44 (0.69, 3.03)
Strong support	16	24	Reference	Reference
**Perceived stigma**				
Yes	20	17	0.69	0.87 (0.43, 1.77)
No	90	88	Reference	Reference
**Lifetime substance use of at least one of khat, cigarette, or alcohol**				
Yes	74	53	0.01	4.96 (2.85, 8.61)
No	36	52	Reference	Reference
**Substance use in the past 3 months of at least one of khat, cigarette, or alcohol**				
Yes	70	48	0.009	4.81 (2.79, 8.31)
No	40	57	Reference	Reference
**Duration of illness**				
<1 year	25	24	Reference	Reference
1–3 years	38	29	0.54	0.79 (0.38, 1.67)
4–6 years	24	23	0.99	0.99 (0.45, 2.22)
7 years and above	23	29	0.49	1.31 (0.60, 2.87)
**Duration of care given**				
<1 month	7	11	Reference	Reference
1 month to 1 year	55	51	0.31	0.59 (0.21, 1.64)
2–3 years	22	15	0.16	0.43 (0.14, 1.37)
4 years and above	26	28	0.49	0.69 (0.23, 2.03)
**Time spent with the patient per day**				
1–6 h	32	24	Reference	Reference
7–12 h	39	30	0.94	1.03 (0.50, 2.09)
Whole day	39	51	0.11	1.74 (8.90, 3.42)
**Number of admissions of patient**				
1 time	25	24	0.70	1.14 (0.584, 2.22)
2 times and above	21	27	0.22	1.52 (7.75, 29.9)
No admission	54	64	Reference	Reference
**Family history of mental illness of caregiver**				
Yes	28	13	0.02	4.10 (2.01, 8.52
No	82	92	Reference	Reference
**Family history of suicide of caregiver**				
Yes	16	9	0.18	5.50 (2.30, 13.10)
No	94	96	Reference	Reference
**History of suicidal attempt of patient**				
Yes	20	25	0.31	1.41 (0.73, 2.72)
No	90	80	Reference	Reference
**Diagnosis of patient**				
Psychosis	52	28	0.01	3.00 (1.20, 7.70)
Bipolar disorder	29	31	0.29	0.60 (0.23, 1.57)
Depression	20	30	0.74	0.84 (0.31, 2.28)
Anxiety disorder	9	16	Reference	Reference

* *Separated, widowed*.

** *Daily laborer, private business, housewife*.

### Multivariable Logistic Regression Analyses of Factors Associated With Common Mental Disorders Among Informal Primary Caregivers of Adults With Any Mental Illness

The AOR of experiencing CMDs among female informal primary caregivers was relatively higher compared with male informal primary caregivers of adult persons with mental illness (AOR 1.98, 95% CI: 1.05, 3.76). Informal primary caregivers who were students by occupation were found to have higher odds ratio of experiencing CMDs compared with government employer caregivers (AOR 5.80, 95% CI: 1.20, 28.40). The AOR of developing CMDs among informal primary caregivers of adults with psychosis was higher compared with the informal primary caregivers of adults with an anxiety disorder (AOR 3.33, 95% CI: 1.12, 9.92), and also, the informal primary caregivers of adults with bipolar disorders were found to have higher AOR of experiencing CMD compared with the informal primary caregivers of adults with anxiety disorders (AOR 3.12, 95% CI: 1.35, 7.23).

Variables including informal history of lifetime and current (during the past 3 months) substance use at least one of khat, cigarette, or alcohol of the primary caregivers, spending the whole day time with the patient, family history of suicide of the caregiver, caring for patients who have two times and above admission history, and family history of mental illness of the caregiver had a statistically significant association with CMDs among informal primary caregivers of adults with mental illness during bivariable logistic regression analyses at *p*-value < 0.25. However, in the final multivariable model, after we adjusted for other variables, they have no association with CMDs (Table 4).

**Table 4:** Multivariable logistic regression analysis of factors associated with CMDs among primary informal caregivers of adults with mental illness at the HFSUH, Ethiopia, 2020.

**Variables**	**Common mental disorder**		***p*-value**	**Adjusted odds ratio (AOR) (95% CI)**
	**Yes**	**No**		
**Sex of the caregivers**				
Male	58	37	Reference	Reference
Female	52	68	0.04	**1.98 (1.05, 3.76)**
**Occupational status**				
Government employer	19	13	Reference	Reference
Merchant	20	29	0.66	1.30 (0.44, 3.75)
Farmer	40	24	0.05	2.50 (0.99, 6.47)
Student	3	11	0.03	**5.80 (1.2, 28.4)**
Other^**^	28	28	0.47	0.72 (0.29, 1.76)
**Lifetime substance use of at least one of khat, cigarette, or alcohol**				
Yes	74	53	0.75	1.27 (0.28, 5.68)
No	36	52	Reference	Reference
**Substance use in the past 3 months of at least one of khat, cigarette, or alcohol**				
Yes	70	48	0.38	0.51 (0.11, 2.31)
No	40	57	Reference	Reference
**Time spent with the patient per day**				
1–6 h	32	24	Reference	Reference
7–12 h	39	30	0.86	1.08 (0.47, 2.5)
Whole day	39	51	0.11	1.91 (0.87, 4.19)
**Number of admissions**				
1 time	25	24	0.22	1.62 (0.75, 3.52)
2 times and above	21	27	0.31	1.51 (0.69, 3.32)
No admission	64	54	Reference	Reference
**Family history of mental illness of caregiver**				
Yes	28	13	0.52	0.75 (0.32, 1.78)
No	82	92	Reference	Reference
**Family history of suicide of caregiver**				
Yes	16	9	0.12	0.45 (0.16, 1.24)
No	94	96	Reference	Reference
**Diagnosis of patient**				
Psychosis	52	28	0.03	**3.33 (1.12, 9.92)**
Bipolar disorder	29	31	0.008	**3.12 (1.35, 7.23)**
Depression	20	30	0.07	2.12 (0.95, 4.73)
Anxiety disorder	9	16	Reference	Reference

** *Daily laborer, private business, housewife. Bold values indicate variables significantly associated with CMDs*.

## Discussion

The current study was designed to assess the prevalence and factors associated with CMDs among the informal primary caregivers of adults with any mental illness during the Covid-19 epidemic and who were attending mental health care service at HFSUH, Eastern Ethiopia.

This study revealed that during the Covid-19 epidemic, the overall prevalence of the CMDs among 215 informal primary caregivers of adults with mental illness was found to be 40.50% with a range between 36.66 and 44.34%. In the final multivariable logistic regression analyses model, being a female caregiver, being a student caregiver, and caring adults with psychosis and bipolar disorders were found to have a significant association with CMDs among informal primary caregivers.

In our study, the prevalence of the CMDs among informal primary caregivers of adults with any mental illness was higher than the results of many studies conducted pre-Covid-19 times: 19.00, 27.10, 31.50, and 33.60%, respectively (26, 27, 41, 42). This discrepancy could be most probably due to the possible Covid-19 pandemic impact. Therefore, the readers can imagine how much it will be challenging to the informal primary caregivers of individuals with mental illness being in the Covid-19 pandemic because both caring for mentally ill patients and the pandemic are different challenging situations that could worsen the physical, psychological, social, and economic status of the caregivers. The other possible reason for the above discrepancy could be the difference in data collection tools and study designs. A previous study (41) used Kessler's K10 to assess the psychological distress/common mental disorder of the caregivers, while in the current study, we used the SRQ − 20 questionnaire to assess the CMDs of the caregivers. Also, the other previous study (42) was community based and our current study was hospital based among primary informal caregivers of adult individuals with mental illness, and this result revealed that caring for persons with mental illness will lead caregivers to be more distressed and develop CMDs than the general population (43).

However, the prevalence of CMDs among informal primary caregivers of adults with any mental illness was closer to the findings in Mexico (40.00%), which was reported pre-Covid-19 time (17), and also lower compared with studies conducted in Ethiopia among caregivers of individuals with severe mental illness at Amanuel Hospital, which reported that the magnitude of mental distress was 56.70% (25), and in Pakistan, among caregivers of people with schizophrenia, which revealed that 72% of caregivers had psychological distress (44). This difference could be due to the diagnosis of the patients. For instance, the studies in Pakistan and in Ethiopia at Amanuel Hospital were conducted among primary informal caregivers of individuals with severe mental illness like schizophrenia, which is the most severe type of mental disorder, and its clinical features, especially positive symptoms, could stress and frustrate caregivers more, and this can increase the prevalence of CMDs among caregivers (43). However, in our study, different diagnoses of patients, including relatively the less severe form of mental disorders (anxiety disorder) to the most severe form of psychiatric disorders (psychosis), were included (43).

In our study, female informal primary caregivers were found to have higher AOR of developing CMDs than male informal primary caregivers (AOR 1.98, 95% CI:1.05, 3.76), and this result was in line with the previous study findings reported both during the Covid-19 pandemic and pre-Covid-19 times (25, 29, 42). The explanations for this variance could be hormonal differences and behavioral models of learned helplessness (43). Previous studies indicated that stress exposure particularly in females would give rise to mental problems, and females are also more susceptible to insomnia. Thus, the female gender might be a higher risk factor for depression and anxiety symptoms (45).

In this study, the primary informal caregivers who were students by occupation were found to have higher AOR of developing CMDs compared with the primary caregivers who were government employers (AOR 5.80, 95% CI:1.20, 28.40). Even if we could not get an available study concerning this issue, this result could be due to many burdens on the student caregivers including time burden, role burden, emotional burden, and financial burden including affording personal protective equipment during the Covid-19 epidemic and, also, school absenteeism due to caregiving and its consequences like academic crisis, which could increase the prevalence of CMDs among primary caregivers who were students.

The current study revealed that the informal primary caregivers who were caring for people with psychosis (AOR 3.33, 95% CI:1.12, 9.92) and bipolar disorders (AOR 3.12, 95% CI:1.35, 7.23) were found to have higher AORs of experiencing CMDs compared with the informal primary caregivers who were caring for adults with an anxiety disorder. This finding was supported by the previous studies conducted both during the Covid-19 pandemic and pre-Covid-19 time (10, 18, 25, 46), and this finding could be due to people, with the diagnosis of severe mental disorders like schizophrenia, exhibiting more positive (difficult behaviors) or negative symptoms, low functioning, and also less likely had to get improvement than other mental disorders like anxiety disorder and depression. Therefore, caring for someone with severe mental disorders can compromise the own physical and psychological health of the caregiver, and can lead to psychological distress, anxiety, depression, and posttraumatic stress disorder (47).

## Limitations of the Study

There are some limitations to this study. Since the study design was cross-sectional, it cannot show the causal relationship. It is difficult to determine whether the CMDs of caregivers existed before the endemic or resulted from it. Besides, since the sample was only taken from informal primary caregivers of patients who came to the hospital, the findings may not represent the informal primary caregivers who stayed at home or in the community, and we cannot generalize all informal primary caregivers of adults with mental illness. The study variables included were not comprehensive enough, and we had missed some important Covid-19-related variables like longer media exposure to get the Covid-19 update.

## Conclusion

This study finding showed a relatively high prevalence of CMDs among informal primary caregivers of adults with any mental illness compared with the previous available studies conducted pre-Covid-19 times (26, 27) and also relative to the study conducted among the general population (42). Factors including being a female caregiver, being a student caregiver, and caring for adults with psychosis and bipolar disorders had a significant association with CMDs among informal primary caregivers of adults with mental illness. This study finding indicates that policymakers and mental health professionals have to give value to the health of informal primary caregivers of persons with mental illness especially during this Covid-19 pandemic, and they have to screen routinely and provide them with appropriate treatments, which might range from providing psycho-education to physical treatments. Additionally, the governments and policymakers should promote the establishment of self-help groups of informal primary caregivers of individuals with mental illness within the community in liaison with social workers/mental health workers, if possible, in which emerge specific modes of social support to help each other and has a key role in the prevention, early diagnosis, and treatment of caregivers who may exhibit the need for mental health care. Our study findings will also serve as the baseline information for further study.

## Data Availability Statement

The original contributions generated for the study are included in the article/supplementary material, further inquiries can be directed to the corresponding author/s.

## Ethics Statement

The studies involving human participants were reviewed and approved by Institutional Health Research Ethics Review Committee (IHRERC) of Haramaya University, College of Health and Medical Sciences. The patients/participants provided their written informed consent to participate in this study.

## Author Contributions

TA contributed to the conceiving of the original idea, designing and conducting the study, analyzing the data, and preparing, critically reviewing, and approving the manuscript for publication. DD contributed to the designing and conducting of the study, analyzing the data, and reviewing the manuscript. Both authors read and approved the final submitted manuscript.

## Conflict of Interest

The authors declare that the research was conducted in the absence of any commercial or financial relationships that could be construed as a potential conflict of interest.

## Publisher's Note

All claims expressed in this article are solely those of the authors and do not necessarily represent those of their affiliated organizations, or those of the publisher, the editors and the reviewers. Any product that may be evaluated in this article, or claim that may be made by its manufacturer, is not guaranteed or endorsed by the publisher.
